# Orexin Receptor Activation Generates Gamma Band Input to Cholinergic and Serotonergic Arousal System Neurons and Drives an Intrinsic Ca^2+^-Dependent Resonance in LDT and PPT Cholinergic Neurons

**DOI:** 10.3389/fneur.2015.00120

**Published:** 2015-06-02

**Authors:** Masaru Ishibashi, Iryna Gumenchuk, Bryan Kang, Catherine Steger, Elizabeth Lynn, Nancy E. Molina, Leonard M. Eisenberg, Christopher S. Leonard

**Affiliations:** ^1^Department of Physiology, New York Medical College, Valhalla, NY, USA; ^2^Department of Medicine, New York Medical College, Valhalla, NY, USA

**Keywords:** acetylcholine, arousal, brain slice, hypocretin, oscillations, reticular activating system, serotonin

## Abstract

A hallmark of the waking state is a shift in EEG power to higher frequencies with epochs of synchronized intracortical gamma activity (30–60 Hz) – a process associated with high-level cognitive functions. The ascending arousal system, including cholinergic laterodorsal (LDT) and pedunculopontine (PPT) tegmental neurons and serotonergic dorsal raphe (DR) neurons, promotes this state. Recently, this system has been proposed as a gamma wave generator, in part, because some neurons produce high-threshold, Ca^2+^-dependent oscillations at gamma frequencies. However, it is not known whether arousal-related inputs to these neurons generate such oscillations, or whether such oscillations are ever transmitted to neuronal targets. Since key arousal input arises from hypothalamic orexin (hypocretin) neurons, we investigated whether the unusually noisy, depolarizing orexin current could provide significant gamma input to cholinergic and serotonergic neurons, and whether such input could drive Ca^2+^-dependent oscillations. Whole-cell recordings in brain slices were obtained from mice expressing Cre-induced fluorescence in cholinergic LDT and PPT, and serotonergic DR neurons. After first quantifying reporter expression accuracy in cholinergic and serotonergic neurons, we found that the orexin current produced significant high frequency, including gamma, input to both cholinergic and serotonergic neurons. Then, by using a dynamic clamp, we found that adding a noisy orexin conductance to cholinergic neurons induced a Ca^2+^-dependent resonance that peaked in the theta and alpha frequency range (4–14 Hz) and extended up to 100 Hz. We propose that this orexin current noise and the Ca^2+^ dependent resonance work synergistically to boost the encoding of high-frequency synaptic inputs into action potentials and to help ensure cholinergic neurons fire during EEG activation. This activity could reinforce thalamocortical states supporting arousal, REM sleep, and intracortical gamma.

## Introduction

Gamma band oscillations (30–60 Hz) occur in many cortical and subcortical regions, and are observed in both waking and sleep states. Their mechanisms and functions have garnered significant interest, as they are thought to play important roles in several high-level cognitive functions including perceptual binding, visual attention, memory, and decision-making [for review, see Ref. (1, 2)]. Moreover, features of schizophrenia have been linked to altered prefrontal gamma activity and decreased inhibitory function of prefrontal parvalbumin interneurons [for review, see Ref. (3)]. Altered gamma activity may also be involved in other neuropsychiatric disorders (4).

The intracortical mechanisms generating synchronized gamma activity involve synaptic interaction between excitatory cortical pyramidal neurons and fast, synchronized inhibitory input from parvalbumin-containing fast-spiking interneurons [for review, see Ref. (1, 2)]. Indeed, selective activation of parvalbumin interneurons is sufficient to drive local gamma oscillations (5, 6), while their selective inhibition dampens spontaneous gamma activity (6).

Gamma activity is also generated in thalamic neurons (7–10), and expression of cortical gamma is thought to be powerfully influenced by thalamocortical and corticothalamic feedback (11), which, in addition to providing a likely substrate for consciousness (12), plays an important role during early development in driving cortical gamma and establishing thalamocortical topography (13).

Electrical stimulation studies, dating back to Moruzzi and Magoun (14), indicate that the mesencephalic reticular formation suppresses cortical slow wave activity and promotes cortical EEG activation. More recently, it has been shown that this activation promotes intracortical, stimulus-specific firing synchronization in the gamma band (15, 16). Combined pharmacological and electrical stimulation studies further suggest that thalamic-projecting mesopontine cholinergic neurons in the laterodorsal (LDT) and pedunculopontine (PPT), particularly through their input to midline thalamic structures, promote gamma band (20–40 Hz) oscillations (7) and foster spatial coherence of these oscillations that outlast the stimulation (17, 18). Thus, the ascending arousal system, including mesopontine cholinergic neurons, appears to play an important role in regulating the expression of forebrain gamma oscillations.

Several reports have recently shown that some ascending arousal system neurons, including all PPT neurons, produce high-threshold, Ca^2+^-dependent oscillations/spikes at gamma frequencies in response to strong depolarization [for review, see Ref. (19, 20)]. These observations have been interpreted to mean that the ascending arousal system is a generator of gamma oscillations. However, it is unclear whether arousal-related inputs to these neurons can generate such intrinsic oscillations or how such oscillations might be transmitted to neuronal targets.

Hypothalamic orexin (hypocretin) neurons provide important arousal-promoting, excitatory input to the entire ascending arousal system and midline and intralaminar thalamic nuclei (21). In cholinergic LDT/PPT neurons and serotonergic dorsal raphe (DR) neurons, orexin peptides produce a slow depolarization that is mediated by an unusually noisy cation current (22–27). Here, we first investigated whether this noisy current can provide significant high frequency input to these neurons, and then whether such high-frequency input can engage Ca^2+^ dependent oscillations in cholinergic LDT and PPT neurons.

Our findings indicate that the noisy orexin current generates significant high frequency, including gamma band, input to both cholinergic and serotonergic neurons, and that cholinergic LDT and PPT neurons have a broad Ca^2+^-dependent resonance that peaked in the theta to alpha frequency range (4–14 Hz) and extended up to 100 Hz.

## Materials and Methods

All procedures complied with NIH guidelines and were approved by New York Medical College Institutional Animal Care and Use Committee.

### Mice

Mice used in the study were C57Bl6 (Taconic), ChAT-IRES-Cre (006410, Jackson; ChAT-Cre), FEV-Cre (012712, Jackson; also known as ePet-Cre), and Cre-inducible reporter mice making EYFP (006148, Jackson), ChR2-EYFP (012569, Jackson), or tdTomato (007909, Jackson; dTom). Cre-reporter mice were bred with ChAT-Cre and FEV-Cre mice to visualize cholinergic neurons and serotonergic neurons, respectively. We first estimated the efficacy and specificity of reporter fluorescence expressed in mesopontine cholinergic neurons from ChAT-Cre mice (28) using each of the above reporter mice. We then quantified the efficacy and specificity of reporter fluorescence in DR serotonergic neurons in offspring from FEV-Cre mice (29) bred with the dTom reporter. Offspring of dTom reporter mice crossed with ChAT-Cre and FEV-Cre mice were then used for brain slices.

### Immunofluorescence

Mice (6–12 weeks) were anesthetized with isofluorane and then overdosed with ketamine (90 mg/Kg)/xylazine (12 mg/Kg) and perfused through the heart with heparinized physiological saline (50 ml) followed by 4% paraformaldehyde (PFA). Brains were removed, post-fixed in 4% PFA for at least 24 h, and then cryoprotected with 30% sucrose in PBS. A one-in-four series of free-floating frozen sections (30 or 40 μm) were incubated on a shaker table overnight at room temperature with primary antibodies. For the ChAT-Cre offspring, cells were labeled with antibodies against ChAT (goat anti-ChAT polyclonal, Millipore, AB144P-200, 1:400), GFP (rabbit polyclonal, Life Technologies, A6455, 1:400) to detect EYFP, or both ChAT and GFP, while the fourth series were untreated. These primary antibodies were visualized with appropriate Alexa-labeled secondary antibodies (GFP: donkey anti-rabbit, Alex-488, Life Technologies, A21206, 1:500; ChAT: donkey anti-goat; Alexa-594, Life Technologies, A11058, 1:500). Sections destined for double labeling were first incubated in GFP antibody and treated with secondary antibody Alexa-488, then incubated with ChAT antibody for another 24 h at room temperature, prior to treatment with Alexa-594-labeled secondary antibody. Sections were then mounted, dehydrated, cleared in xylene, and coverslipped with non-fluorescent mounting medium (Krystalon, EMD, 64969-95).

Two control experiments for double labeling were conducted. In the first, secondary antibodies were mismatched with the primary to determine if there was any labeling of the GFP by the anti-ChAT secondary or if there was any anti-ChAT labeling by the anti-GFP secondary. In the second control, primary antibodies were omitted to determine if there was any direct tissue labeling by the secondary antibodies. Neither control produced any labeling above background.

For offspring of ChAT-Cre and dTom reporter mice, a one-in-four series of free-floating frozen sections were processed with the ChAT antibody as above but visualized with donkey anti-goat; (Alexa-488, Life Technologies, A-11055, 1:500). Sections were mounted and then coverslipped with Vectashield HardSet (Vector Labs), since the dTom fluorescence was sufficiently stable and bright to directly detect.

For offspring of FEV-Cre and dTom reporter mice, a one-in-four series of free-floating frozen sections were processed with a TPH antibody (sheep anti-TPH polyclonal; BioLegend 816401, formerly Covance PSH-327P, 1:400) visualized with donkey anti-sheep; (Alexa-488, Life Technologies, A-11015, 1:500), and the sections were mounted and then coverslipped with Vectashield HardSet (Vector Labs).

### Quantification of expression

ChAT immunolabelled sections were inspected and photographed using a photomicroscope (BX60, Olympus) with an epifluorescence illuminator (100 W Hg arc-lamp) and a digital camera (QIC-F-M-12-C, Q-imaging) operated with iVision software (Biovision) running on a Mac OS computer. Alexa-488 was visualized using a Chroma 41001 filter cube and Alexa-594 and dTom were visualized with a Chroma 41004 filter cube. Single labeled sections were inspected first and then double labeled sections were quantified. Double-labeled sections were photographed with a 4× objective and then a field within either the LDT or PPT was selected and photographed using a 10× objective for EYFP and ChAT for counting. Each identifiable cell was examined for each fluorophor and counted as ChAT+, EYFP+, or ChAT+/EYFP+. In the first series of mice (*n* = 5), which were offspring from the ChAT-Cre x EYFP reporters, a single region from each animal was counted in the middle of LDT (total of 550 cells) and PPT (total of 395 cells). In the second series of mice (*n* = 3), which were offspring from the ChAT-Cre x ChR2-EYFP reporter, a single region from each animal was counted in the caudal and middle LDT (total of 243 cells) and the middle and rostral PPT (total of 234 cells). In a third series of mice (*n* = 2), we immunolabelled offspring from a ChAT-Cre x dTom reporter and counted two LDT (total of 291 cells) and two PPT (total of 172 cells) regions from each animal.

Since cell density was substantially higher in parts of the DR, quantification of the FEV-Cre, dTom mice (*n* = 3) were conducted using Z-series image stacks acquired with a Zeiss LSM 710 confocal microscope. Alexa-488 and dTom fluors were excited with 488 nm argon and 543 nm helium-neon lasers, respectively. A 10× objective was used for imaging of both fluors obtained from identical *Z* axis series, with the Z-positioning based on a pinhole diameter of 1 Airy-Unit calibrated according to dTom fluorescence. The DR was divided into three regions (lateral, dorsal, and ventral) and cells were counted within each region from two sections per animal separated by 160 μm beginning approximately just in front of the rostral pole of the LDT. Each identifiable cell in the stack within the region was counted as TPH+, dTom+, or TPH+/dTom+. A total of 3409 DR neurons were counted (lateral: *n* = 819; dorsal: *n* = 471; ventral: *n* = 824).

Cell counts from wide-field images and confocal image stacks were accomplished using ImageJ software^1^ and the Cell Counter plug-in (Kurt De Vos^2^, University of Sheffield).

### Brain slice preparation

Mice (*n* = 26), ages P15–P31 (plus one mouse P10), were decapitated following induction of deep anesthesia with isofluorane. A block of brain with the target structure was rapidly removed and incubated, and then cut in ice-cold artificial cerebrospinal fluid (ACSF) which contained (in mM) 124 NaCl, 5 KCl, 1.2 NaH_2_PO_4_, 2.7 CaCl_2_, 1.2 MgSO_4_, 26 NaHCO_3_, and 10 dextrose (295–305 mOsm), and was oxygenated with carbogen (95% O_2_ and 5% CO_2_). Brain slices (250 μm) were cut with a Leica vibratome (VT1000S), and were then incubated at 35°C for 15 min in oxygenated ACSF. They were then stored in continuously oxygenated ACSF at room temperature until use. For recording, slices were submerged in a recording chamber that was perfused at 1–2 ml/min with continuously oxygenated ACSF, which was at room temperature (23 ± 2°C) for measurement of the spectral properties of the orexin current noise. For dynamic clamp experiments, ACSF was heated to 32°C with an in-line heater and temperature controller (Warner Instruments; TC-324C).

### Drugs

Ionotropic receptor antagonists 6,7-Dinitroquinoxaline-2,3(1H,4H)-dione (DNQX, 15 μM, Sigma), D(-)-2-Amino-5-phosphonopentanoic acid (AP5, 50 μM, Sigma, Tocris Bioscience), SR-95531 (Gabazine, 20 μM, Sigma) with strychnine (2.5 μM, Sigma) were added to ACSF in all experiments to block fast synaptic potentials. Additionally, tetrodotoxin (TTX, 500 nM, Alomone Labs) was added to block voltage-gated sodium channels. Orexin-A (Peptide international, USA; 300 nM) was diluted into ACSF to final concentration from frozen aliquots just before use.

### Whole-cell electrophysiological recording and imaging

Whole-cell recordings (seals >3 GΩ) were obtained from neurons in the LDT, PPT, and DR as previously described (25). Briefly, borosilicate micropipettes (2–4 MΩ; cat # 8050, AM systems) were utilized and neurons were visualized for whole cell recordings at 160× magnification using visible-light, differential interference contrast (DIC) optics and a Nuvicon tube camera (Dage VE-1000) mounted on a fixed stage microscope (Olympus BX50WI). Neurons were selected for patching based on their DIC image after confirming expression of dTomato fluorescence captured with a Micromax camera (Roper Scientific) that was controlled with custom software (TIWB; see below).

The pipette solution contained (in mM): 144 K-gluconate, 3 MgCl_2_, 10 HEPES, 0.3 NaGTP, and 4 Na_2_ATP (310 mOsm) and *bis*-fura 2 (50 μM; Invitrogen) was added for calcium chelation and imaging. Biotinylated Alexa-594 (25 μM; Invitrogen) was also included in all experiments for cell identification. This allowed us to visualize both the pipette and target cell dTomato fluorescence simultaneously. Gigaseals were obtained using an Axopatch 200B amplifier (Molecular Devices) operated in voltage clamp mode, filtered at 2 or 5 KHz, and sampled at 20 KHz.

To lower extracellular calcium concentration and to block voltage-gated Ca^2+^ channels, Co^2+^ was substituted for Ca^2+^ in the ACSF, which contained (in mM) 125.25 NaCl, 5 KCl, 2.7 CoCl_2_, 1.2 MgCl_2_, 26 NaHCO_3_, and 10 dextrose. Cobalt was added to this ACSF just before superfusing.

Whole cell recordings were initiated in voltage clamp mode following pipette capacitance compensation. After breakthrough, a brief series of voltage protocols were delivered to ascertain the presence of A-type K^+^ currents and/or T-type Ca^2+^ currents for LDT and PPT cholinergic neurons (30–32) and a strong medium AHP current for DR serotonergic neurons [see Ref. (33)]. Voltage clamp mode was used to record orexin currents in order to assess their spectral characteristics. For dynamic clamp experiments (see below), the amplifier was switched to current clamp (fast) mode. The quality of the recording was assayed by monitoring holding current, access resistance, and input resistance as determined by responses to brief, negative going steps. These parameters were monitored throughout the recording and the experiment was terminated if estimated access resistance became unstable or changed by more than ~10% between measurements. Recordings were also terminated if cell parameters became unstable.

Whole cell current and voltage traces were digitized and command pulses were generated with custom software [TIWB; (34)] run on a Mac OS computer controlling an ITC-18 interface (Instrutech-HEKA). In some recordings (Figure 3C), simultaneous changes in somatic Ca^2+^-dependent *bis*-fura 2 fluorescence were also monitored (delta F/F), using TIWB software to control image acquisition and the light-source shutter as we have done previously [see Ref. (25)]. A continuous, ‘Gap-Free’ record of whole-cell currents was obtained using PCLAMP 8 software (Molecular Devices) running on a PC using a Digidata 1322A (Molecular Devices). Current traces were low pass filtered at 2 KHz and sampled at 5 or 10 KHz.

### Dynamic clamp

To deliver a defined noisy orexin current, a virtual orexin conductance (vGorx) was introduced to cells recorded in whole-cell current-clamp (fast) mode by a dynamic clamp [for review, see Ref. (35, 36)]. The dynamic clamp was implemented using QuB software, as modified by Dr. Lorin S. Milescu (37) running on a PC (ASL, Marquis M517-T) controlling a National Instruments multifunction PCIe card (NI PCIe-6251). The virtual orexin conductance was derived from a typical orexin current recorded at −65 mV from a DR neuron having an average conductance of 0.5 nS and an estimated reversal potential of −15 mV. The conductance fluctuations were computed from normalized current fluctuations. The injected noisy virtual orexin current was then determined by the equation Iorx = Gave × Gfluct × (Vm − Vrev), where Gave ranged between 0.5 and 16 nS, Gfluct is the normalized noisy conductance wave, Vm is the membrane potential, and Vrev is the orexin reversal potential (−15 mV). We also compared the effects of this noisy conductance to the effects of a virtual orexin conductance without noise in which Gfluct was replaced by its average value (~1).

### Data analysis

Data were analyzed and figures prepared using Igor Pro 6 (Wavemetrics, Lake Oswego, OR, USA). All reported values of Vm were corrected by −15 mV to compensate for the liquid junction potentials, which were measured for patch solution and ACSF combinations (−15.6 mV). Experimental results are reported as mean ± SEM and were compared using *t*-tests, ANOVAs, or repeated measures multiple ANOVA (rMANOVA; Data Desk 7, Data Description, Inc.) with significance set at *p* < 0.05. Spectral analysis was done using the Sonogram and Power Spectral Density procedures in Igor Pro. Frequency bands were defined as delta: 1–4 Hz; theta: 4–8 Hz; alpha: 8–14 Hz; beta: 14–30 Hz, and gamma: 30–60 Hz.

## Results

### ChAT-cre mice accurately drive reporter expression in the LDT and PPT cholinergic neurons

**Figure 1:**
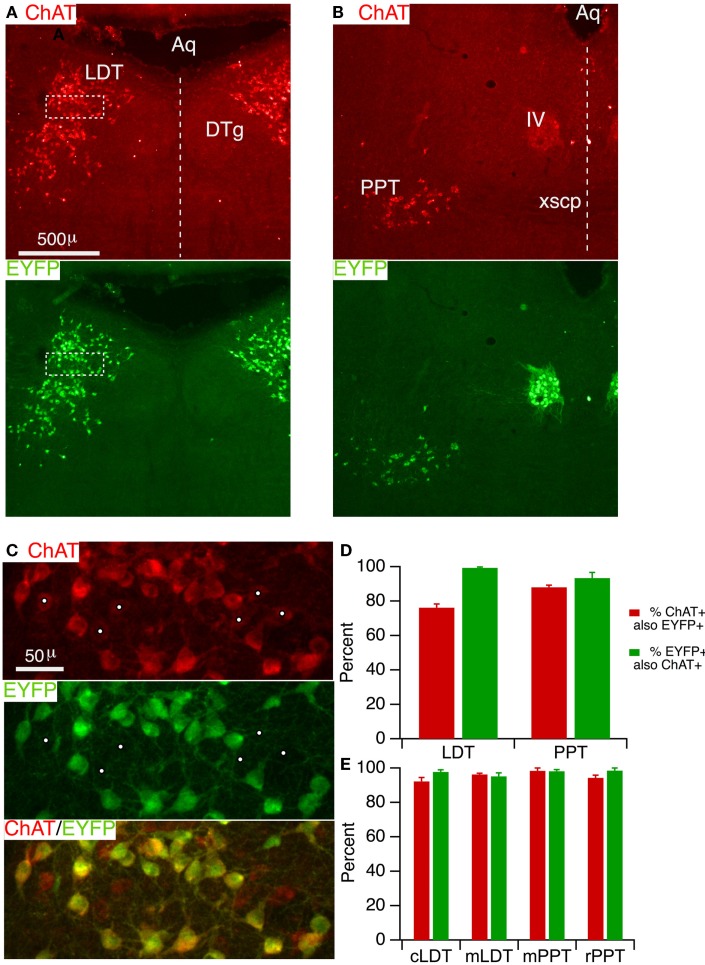
**ChAT-Cre mice accurately drive reporter fluorescence in LDT and PPT cholinergic neurons**. **(A)** Section through the LDT visualized with ChAT immunofluorescence (Alexa 594; top) and EYFP immunofluorescence (Alexa 488; bottom). **(B)** Section through the PPT visualized with ChAT immunofluorescence (Alexa 594; top) and EYFP immunofluorescence (Alexa 488; bottom). **(C)** Higher magnification of the boxed counting region in **(A)**, visualizing label for ChAT (top), EYFP (middle), and both (bottom). The vast majority of ChAT+ neurons was EYFP+. White dots mark faintly labeled ChAT+ neurons, which appeared to lack EYFP. **(D)** Quantifies the efficacy (red) and specificity (green) of YFP reporter fluorescence in labeled cholinergic LDT and PPT neurons (*n* = 5 mice). Efficacy is indicated by the percent of ChAT+ neurons that were also immunopositive for EYFP. Specificity is indicated by the percent of EYFP+ neurons that are also ChAT+. **(E)** Similar quantification of ChAT-Cre expression using a ChR2-EYFP reporter line (*n* = 3 mice). Cells were counted in a field from the caudal LDT (cLDT), middle LDT (mLDT), middle PPT (mPPT), and the rostral PPT (rPPT). Dotted line indicates the midline. Aq, cerebral aqueduct; LDT, laterodorsal tegmental nucleus; DTg, dorsal tegmental nucleus; PPT, pedunculopontine tegmental nucleus; IV, trochlear nucleus; xscp, decussation of the superior cerebellar peduncle.

In order to specifically manipulate neurons through Cre-dependent recombination, a high concordance is necessary between Cre-recombinase expression and phenotypic markers in the population of interest. This has not yet been established in the LDT and PPT cholinergic neurons for the ChAT-Cre driver line utilized in this study (28). We therefore assessed the degree to which Cre-recombinase is correctly expressed in LDT and PPT cholinergic neurons by crossing ChAT-Cre mice with mice harboring a Cre-inducible EYFP reporter. Offspring were then prepared for immunocytochemistry and tissue sections were immunostained for ChAT. Sections taken through the middle of the LDT (Figure 1A, ChAT) showed a large number of ChAT+ (red) neurons extending from the central gray into the underlying tegmental region, as expected (38, 39). Viewing Alexa 488 in the same fields revealed an identical pattern of EYFP expressing neurons, suggesting a high degree of correspondence between ChAT+ and EYFP+ neurons. A similar high correspondence between ChAT and EYFP was observed for sections through the PPT (Figure 1B) as well as in the nearby trigeminal (not shown) and trochlear motor nuclei (Figure 1B). To estimate the efficacy of labeling cholinergic neurons, we counted neurons that were single and double-labeled in a field imaged at higher magnification within the LDT (Figure 1C) and the PPT. This revealed that EYFP was present in the vast majority of ChAT+ neurons. Within the LDT, we found on average (*n* = 5 mice), 76.2 ± 2.1% of ChAT+ neurons also contained EYFP immunoreactivity, while in the PPT we found 88.0 ± 1.2% ChAT+ neurons also contained EYFP immunoreactivity (Figure 1D). Inspection of the counting regions revealed that it was the neurons most weakly labeled for ChAT that appeared negative for EYFP immunoreactivity (Figure 1C, white dots). This suggests that while a large majority of ChAT+ neurons are labeled by reporter fluorescence, a population of weakly ChAT+ neurons may be underrepresented by reporter fluorescence. To assess the specificity with which reporter fluorescence was expressed in cholinergic neurons, we examined the fraction of EYFP+ neurons that were also ChAT+. This revealed that reporter expression was highly specific for cholinergic neurons within these structures (Figure 1D). In the LDT, we found 99.4 ± 0.5% of EYFP+ neurons were ChAT+, while in the PPT, 93.3 ± 3.3% of EYFP+ neurons were ChAT+.

To examine the generality of these findings with a different reporter and to obtain additional information about the efficacy and specificity of Cre-recombinase expression at additional locations within these nuclei, we bred offspring (*n* = 3) from a second cohort of ChAT-Cre mice crossed with ChR2-EYFP reporter mice. In this series, we divided the LDT into a caudal and middle region, and the PPT into a middle and rostral region, and counted single and double-labeled cells from a region centered on the highest density of cholinergic neurons in each section. This cohort revealed an even higher efficacy of cholinergic neuron labeling (Figure 1E), with 92.2 ± 2.2 and 96.2 ± 0.7% ChAT+ neurons containing EYFP in the caudal and middle LDT, and 98.3 ± 1.7 and 94.3 ± 1.5% ChAT+ neurons containing EYFP in the middle and rostral PPT, respectively. Results from this cohort also showed a high degree of specificity with 97.7 ± 1.2 and 95.2 ± 2.0% of EYFP+ neurons containing ChAT in the caudal and middle LDT, and 98.1 ± 1.0 and 98.5 ± 1.5% EYFP+ neurons containing ChAT in the middle and rostral PPT, respectively.

In order to visualize living neurons *in situ* or in brain slices, it is advantageous to use a reporter producing the brightest possible fluorescent proteins, since brighter fluorescence enables visualization with lower intensity illumination and lower phototoxic damage. Moreover, it would be advantageous to use a red-shifted reporter protein, in some cases, to enable simultaneous use of indicator dyes that are excited at ultraviolet and blue wavelengths, like *bis*-fura 2. We therefore crossed a third cohort of ChAT-Cre mice with a recently introduced Cre-dependent reporter mouse expressing tdTomato, which is among the brightest and most photostable fluorescent proteins identified (40), and has been shown to produce much brighter reporter fluorescence than EYFP following Cre recombination (41). While recognizing that reporter efficiency can vary, these reporter mice have been successfully utilized with a large number of Cre-driver lines, including the ChAT-Cre line we used here (41). We therefore checked two ChAT-Cre × dTom offspring and found a comparable efficacy (LDT: 88.4 ± 3.5%; PPT 88.9 ± 3.7%) and specificity (LDT: 91.2 ± 1.6%; PPT 88.5 ± 3.7%) to the other reporters.

Collectively, we found that these ChAT-Cre mice effectively drive reporter fluorescence expression in the LDT and PPT, and that a very large majority of neurons containing reporter fluorescence are cholinergic. Thus, offspring from these ChAT-Cre and fluorescent reporter mice are suitable for studying mesopontine cholinergic neurons.

### FEV-cre drives dTom expression and enables visualization of most serotonergic DR neurons

**Figure 2:**
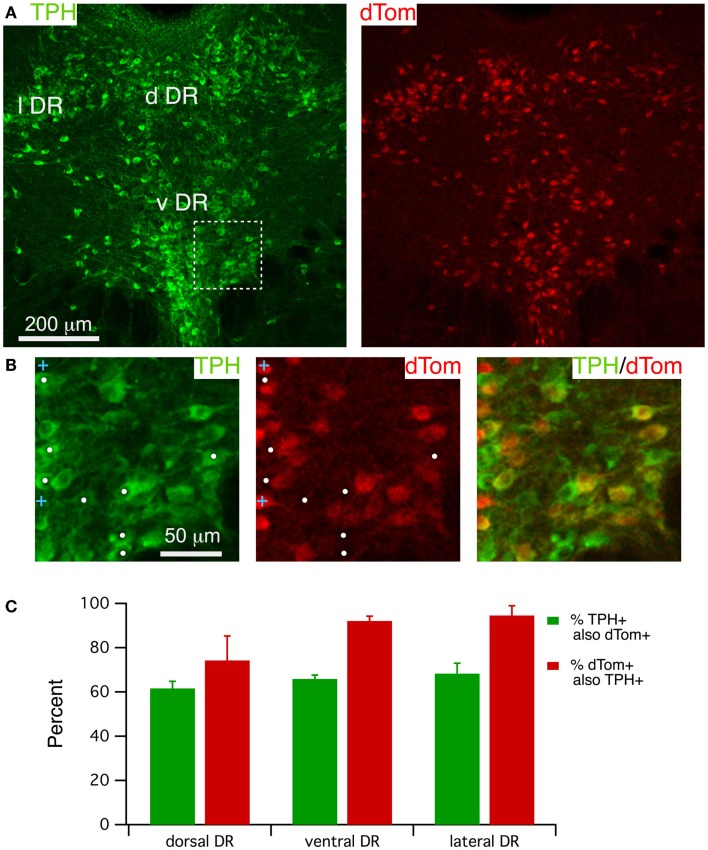
**FEV-Cre mice drive dTom reporter fluorescence in most TPH**+** dorsal raphe neurons**. **(A)** A single confocal section from a Z-stack through the DR visualized with TPH immunofluorescence (488 excitation; left) and dTom reporter fluorescence (543 excitation, right). **(B)** Higher magnification of the boxed region in **(A)**, visualizing label for TPH (left), dTom (middle), and both (right). White dots mark TPH+ but dTom− neurons. Blue crosses mark dTom+ but TPH− neurons. **(C)** Quantification of efficacy (green) and specificity (red) of dTom fluorescence in TPH+ neurons from FEV-Cre; dTom mice (*n* = 3). Cells were counted in fields centered on the dorsal DR (d DR), the ventral DR (v DR), and the lateral DR (l DR).

FEV-Cre mice appear to drive accurate reporter expression in serotonergic neurons (29), although we are not aware of any reports quantifying the accuracy of Cre-induced dTom expression in this line. We therefore examined the accuracy of dTom co-localization with immunolabel for TPH (Figure 2) by counting cells within three DR regions (lateral, dorsal and ventral) from three mice. A single confocal image of TPH immunofluorescence and dTom fluorescence (Figure 2A, left and right; approximately center of the stack) illustrates the extensive co-labeling of TPH+ neurons with dTom, although it is apparent that there are many fewer dTom+ neurons. This is clearer in the expanded images (Figure 2B) from the boxed region in Figure 2A, where the white dots mark TPH+ but dTom− cells in this field. This impression was borne out by cell counts from each region, which indicated that only ~65% of TPH+ cells were also dTom+ (Figure 2C).

It was also apparent that dTom fluorescence was present in some cells lacking TPH immunofluorescence. This was observed mainly in neurons that were smaller than serotonergic neurons, as illustrated by blue crosses in Figure 2B. There was also a trend toward lower specificity within the dorsal region of the DR (74.2 ± 11.0%) compared to the lateral (94.6 ± 4.4%) and ventral (92.1 ± 2.1%) regions (Figure 2C), although this difference did not achieve statistical significance (*p* > 0.1, paired *t*-test). This effect was also variable across mice (specificity range: 52.6–90.1%) and was mainly accounted for by a group of small TPH− cells in the dorsal-most sector of the dorsal DR. Thus, FEV-Cre driven expression of dTom can serve as a good marker for most medium to large TPH + DR neurons, although a substantial number of TPH+ neurons will be unlabeled across all regions of the DR. To reduce the likelihood of recording from dTom expressing TPH− neurons, whole-cell recordings were only obtained from large dTom+ neurons (>25 μm), and we avoided patching neurons in the most dorsal sector of the dorsal DR.

### Genetically identified cholinergic LDT and PPT neurons and serotonergic DR neurons have expected electrophysiological characteristics

**Figure 3:**
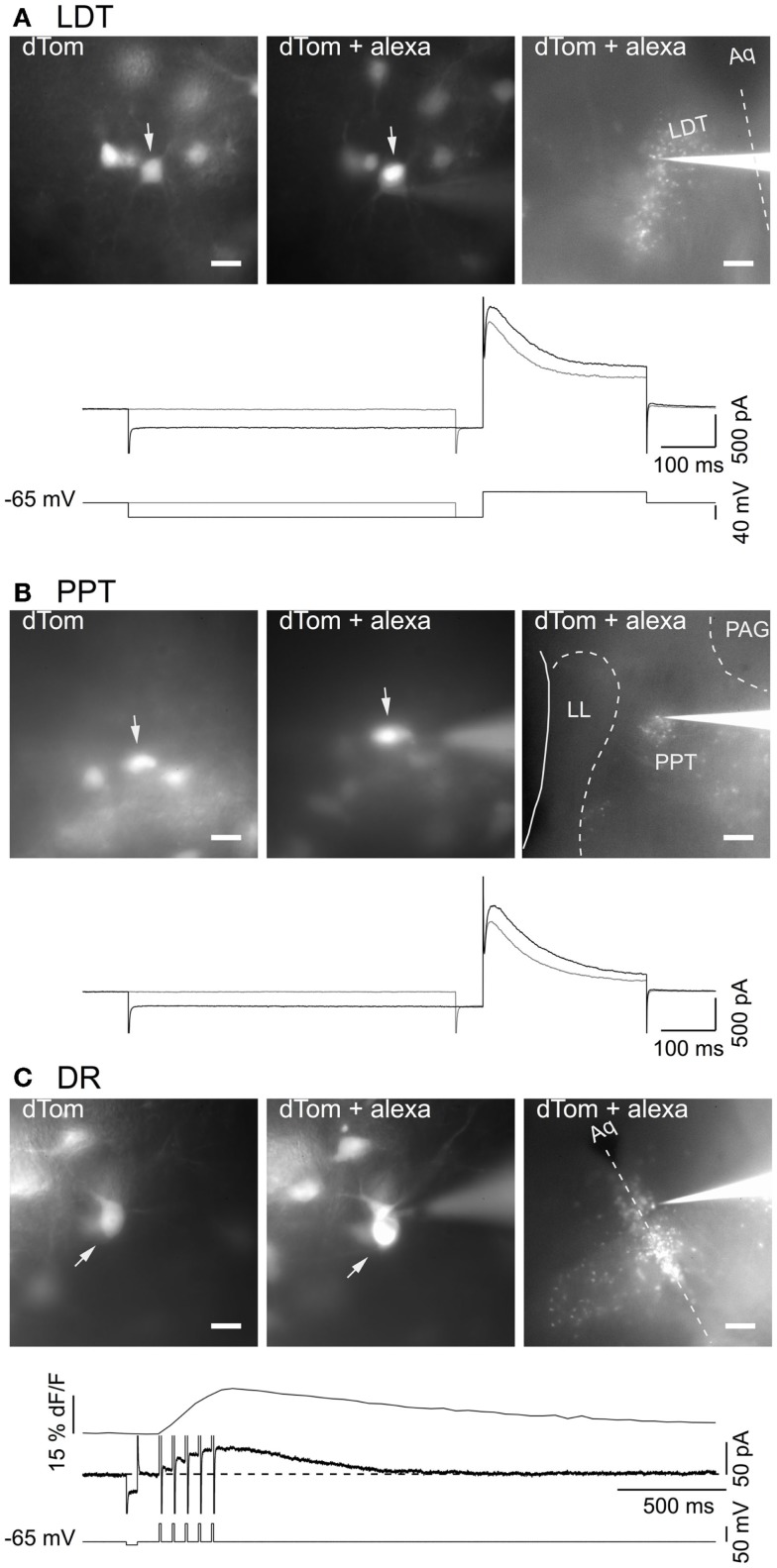
**Genetically identified cholinergic and serotonergic neurons show characteristic membrane currents**. **(A)** Images of *in situ* dTom fluorescence from a group of neurons in the LDT prior to patching the cell marked with an arrow (dTom, left). The same neuron (arrow) was recorded with a patch pipette containing alexa 594 (dTom + alexa; middle). Low power image of the recorded neuron and recording pipette in the field of dTom expressing LDT neurons (dTom + alexa; right). Bottom panel shows the membrane current (middle two traces) elicited by the voltage steps (bottom two traces) and illustrates the transient outward current characteristic of cholinergic LDT neurons. **(B)** Images of dTom fluorescence from a group of PPT neurons prior to patching the cell marked with an arrow (dTom, left). Whole cell recording of the same neuron (arrow) with a pipette containing alexa 594 (dTom + alexa; middle). Low power image of the recorded neuron and pipette in the field of dTom expressing PPT neurons (dTom + alexa; right). Bottom panel illustrates the transient outward current characteristic of cholinergic PPT neurons elicited by the same voltage protocol illustrated in **(A)**. **(C)** Images of dTom fluorescence from a group of DR neurons prior to patching the cell in the center (dTom, top). Whole cell recording of the same neuron with a pipette containing alexa 594 (dTom + alexa; middle). Low power image of the recorded neuron and pipette in the field of dTom expressing DR neurons (dTom + alexa; bottom). Dotted line indicates the midline. Bottom panel illustrates the characteristic somatic Ca^2+^ transient (top trace) measured as delta F/F (dF/F) and medium duration after hyperpolarization current (middle trace) produced by five brief depolarizing pulses (10 ms, 20 Hz) to +10 mV (bottom trace). Scale bars are 20 μm in the left images and apply to the middle images. Scale bar in the right images are 200 μm. Aq, cerebral aqueduct; dF/F, LDT, delta laterodorsal tegmental nucleus; LL, nuclei of the lateral lemniscus; PAG, periaqueductal gray; PPT, pedunculopontine tegmental nucleus.

**Figure 4:**
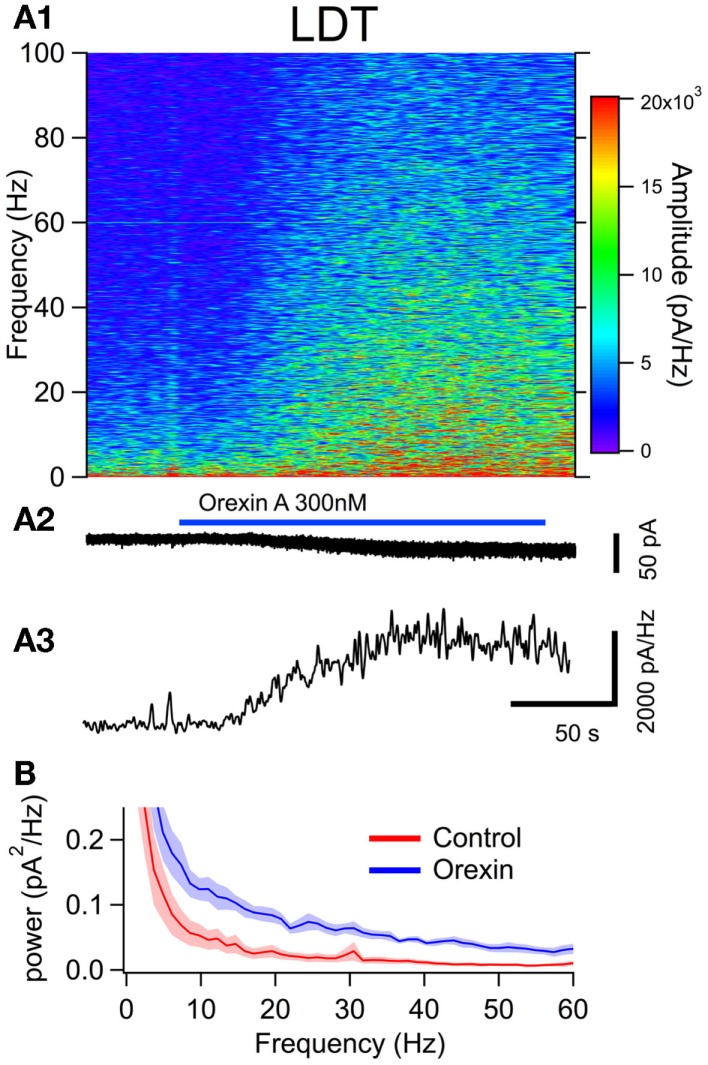
**The orexin current increased membrane current noise in the gamma band of cholinergic LDT neurons**. **(A)** Orexin-A induced a noisy depolarizing (inward) current in LDT cholinergic neurons with substantial power at gamma frequencies. **(A1)** Spectral composition of the membrane current (vertical axis) as a function of time (horizontal axis). Amplitude of the spectrum is displayed according to the color scale (right). **(A2)** shows the membrane current from which the spectrum in **(A1)** was computed. As the orexin-induced inward current developed, there was a pronounced increase in high frequency components of the membrane current extending beyond 100 Hz. **(A3)** shows the time course of the gamma band amplitude. **(B)** The average (±SEM) power spectral density function measured over 30 s before (red; Control) and during the orexin-A action (blue; Orexin; *n* = 4). Orexin produced an increase in power that was distributed over a broad band of frequencies rather than being focused at particular frequencies.

To visualize fluorescent cholinergic and serotonergic neurons in brain slices for patch clamping, we utilized offspring from dTom reporter mice crossed with the ChAT-Cre and FEV-Cre mice. Slices made from these mice had brightly labeled neurons in the LDT, PPT, and DR that were readily selected for whole-cell recording (Figure 3). From a field of labeled neurons within the LDT (Figure 3A, 40× objective: left image, dTom), PPT (Figure 3B, 40× objective: left image, dTom), or DR (Figure 3C, 40× objective: left image, dTom), single neurons were patch-clamped and filled with alexa-594 (Figures 3A–C, middle image, dTom + alexa). Cholinergic neurons recorded in this way had electrical properties expected from previous descriptions of LDT and PPT nNOS+ neurons, with a prominent A-current either alone (type II, Figures 3A,B, bottom traces), or in combination with a T-type Ca^2+^ current (not shown) (30, 31, 42). Similarly, all of our patched neurons from dTom, FEV-Cre mice had the characteristic medium AHP current and prominent somatic Ca^2+^ transient evoked by a train of five brief depolarizing pulses (Figure 3C, bottom traces). Following the experiment, with the electrode still attached to the recorded neuron, a low power image revealed the location of the recorded cell within the LDT, PPT, or DR (Figures 3A–C, 4× objective, right image, dTom + alexa).

### The depolarizing orexin current generates a high frequency input to cholinergic LDT and PPT neurons and 5-ht dorsal raphe neurons

**Figure 5:**
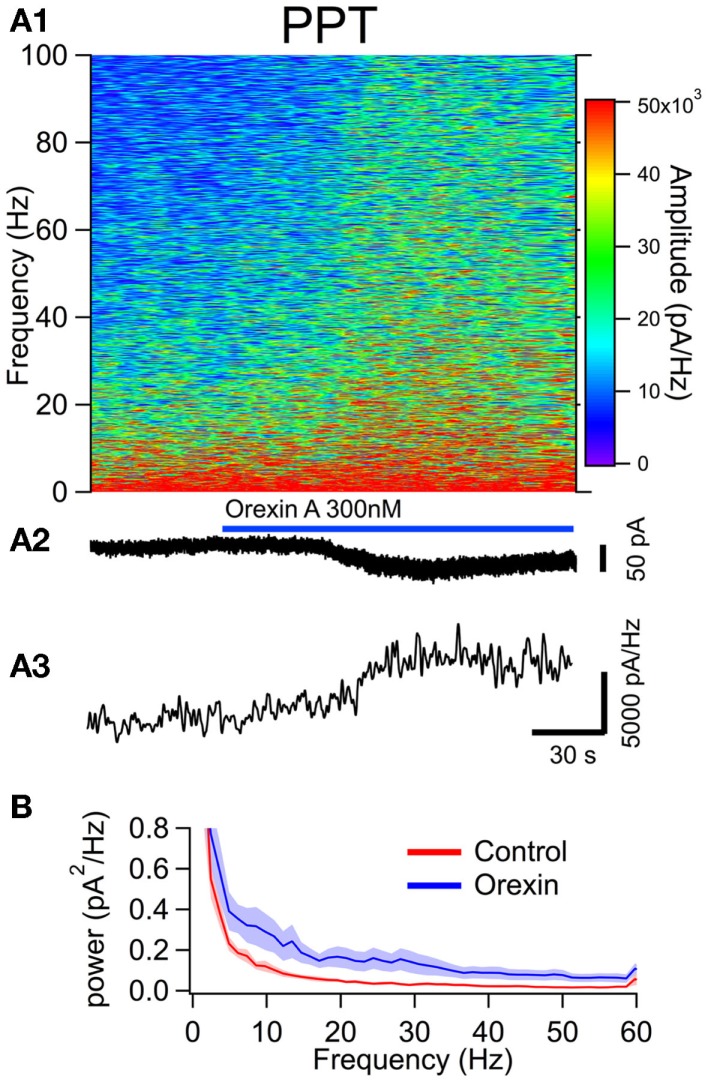
**The orexin current increased membrane current noise in the gamma band of cholinergic PPT neurons**. **(A)** Orexin-A induced a noisy depolarizing (inward) current in PPT cholinergic neurons with substantial power at gamma frequencies. **(A1)** Spectral composition of the membrane current (vertical axis) as a function of time (horizontal axis). Amplitude of the spectrum is displayed according to the color scale (right). **(A2)** shows the membrane current from which the spectrum in **(A1)** was computed. As the orexin-induced inward current developed, there was a pronounced increase in high frequency components of the membrane current extending beyond 100 Hz. **(A3)** shows the time course of the gamma band amplitude. **(B)** The average (±SEM) power spectral density function measured over 30 s before (red; Control) and during the orexin-A action (blue; Orexin; *n* = 4). As in LDT neurons, orexin produced an increase in power that was distributed over a broad band of frequencies rather than being focused at particular frequencies.

**Figure 6:**
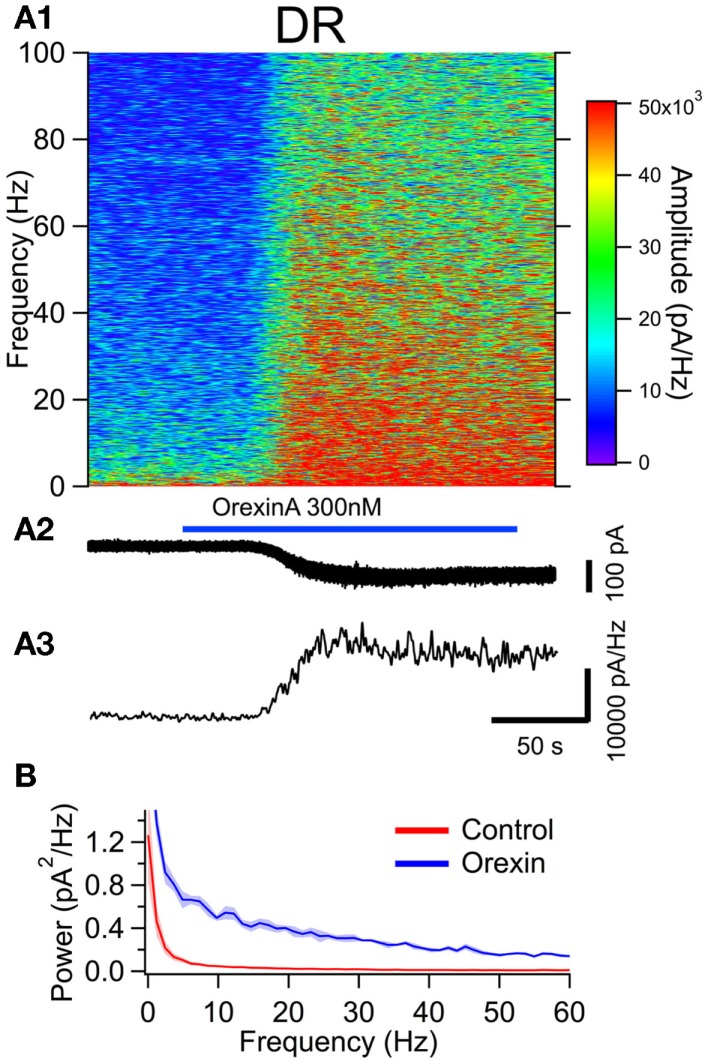
**The orexin current increases membrane current noise in the gamma band of serotonergic DR neurons**. **(A)** Orexin-A induced a noisy depolarizing (inward) current in DR serotonergic neurons with substantial power at gamma frequencies. **(A1)** Spectral composition of the membrane current (vertical axis) as a function of time (horizontal axis). Amplitude of the spectrum is displayed according to the color scale (right). **(A2)** shows the membrane current from which the spectrum in **(A1)** was computed. As the orexin-induced inward current developed, there was a pronounced increase in high frequency components of the membrane current extending beyond 100 Hz. **(A3)** shows the time course of the gamma band amplitude. **(B)** The average (±SEM) power spectral density function measured over 30 s before (red; Control) and during the orexin-A action (blue; Orexin; *n* = 5). Orexin produced an increase in power that was distributed over a broad band of frequencies rather than being focused at particular frequencies.

To examine the effect of orexin on the noise spectrum of identified neurons, we bath applied orexin (300 nM) to dTom fluorescent cholinergic neurons in the LDT and PPT. For a comparison to cholinergic neurons, we conducted parallel experiments in dTom fluorescent serotonergic neurons of the DR. In each of these identified neuronal groups, orexin application produced a slow inward current and an increase in membrane current noise from a holding potential of −65 mV. We then computed the frequency composition of the membrane current as a function of time around the application of orexin (Figures 4A,5A and 6A). In each case, the orexin-evoked inward current was accompanied by a large increase in the amplitude of higher frequency components extending beyond 100 Hz. For example, the increase in the gamma band (30–50 Hz) was substantial and is illustrated for each cell type in panel A3 of Figures 4–6. This demonstrates that in addition to providing a slow depolarizing current, orexin receptor activation in these neurons also provides a high frequency input driven by a spectral shift in the current noise.

**Figure 7:**
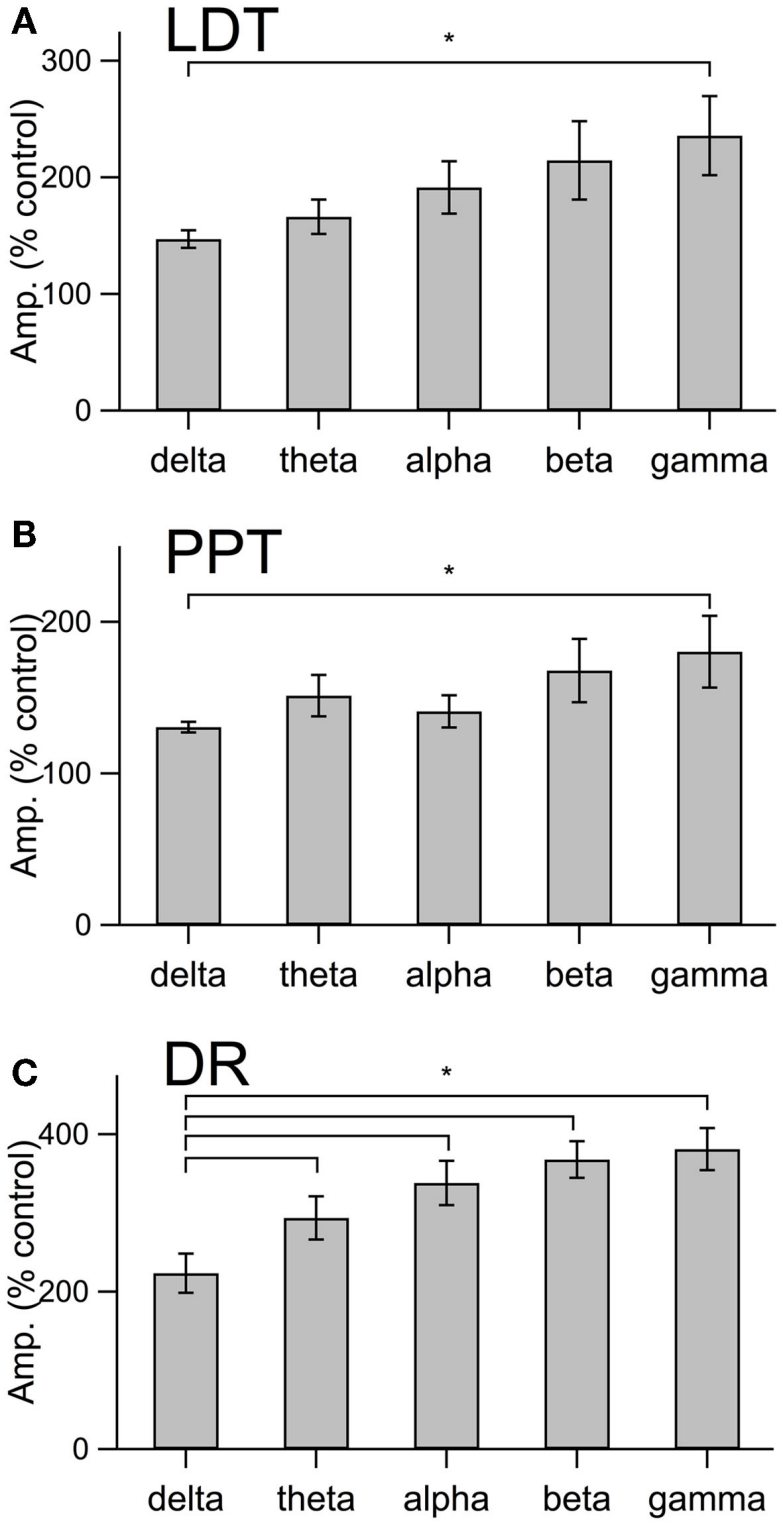
**The orexin current in cholinergic LDT and PPT neurons and in serotonergic DR neurons produce a proportionately greater increase in current at gamma frequencies than at delta frequencies**. **(A–C)** The percent change in membrane current amplitude measured at delta, theta, alpha, beta, and gamma frequency bands for LDT **(A)**, PPT **(B)**, and DR **(C)** neurons. Only the difference between gamma and delta was significant in LDT (*n* = 4) and PPT (*n* = 4) cholinergic neurons. In serotonergic DR neurons (*n* = 5), the difference between the delta band and each other frequency band was significant. *p < 0.05, paired *t*-test.

This increase in noise was not focused into particular frequency bands, but rather constituted a general shift toward higher frequencies. This point is well illustrated in panel B of Figures 4–6, which compares the average (±SEM) power spectral density functions of membrane current from before and during the maximal orexin effect. This shows that the power increase occurs over the entire spectrum without favoring particular frequencies. This can also be seen from the similar increase in amplitude that occurs at key frequencies that change across behavioral state in the EEG (Table 1). However, since the amplitude of membrane current noise falls off steeply with increasing frequency under control conditions, the percent increase over control produced by orexin was larger at higher frequencies (Figure 7). In both LDT and PPT cholinergic neurons, the percent increase in the gamma band was significantly larger than the percent increase in the delta band (*p* < 0.05, paired *t*-test; other frequencies, not different from delta). In the DR, percent increase in the theta, alpha, beta, and gamma bands was significantly larger than the percent increase in the delta band (*p* < 0.05, paired *t*-test).

**Table 1:** **Orexin induces a noisy current with components across a wide range of frequencies in cholinergic LDT (*n* = 4) and PPT (*n* = 4) neurons and serotonergic DR neurons (*n* = 5)**.

	LDT		PPT		DR	
	
	Control	OrexinA	Control	OrexinA	Control	OrexinA
Delta	11669.2 ± 3648.8	16457.6 ± 4666.3*	7092.8 ± 1688.8	28529.8 ± 6149.1*	8719.6 ± 1347.1	19162.5 ± 3865.8*
Theta	7567.6 ± 2327.5	11682.7 ± 3012.7**	12535 ± 2631.9	19037.7 ± 3984.2*	5505.8 ± 821.8	16063.6 ± 3082.1**
Alpha	4509 ± 1460.6	7962.1 ± 2050.6**	10698.4 ± 2476	15195.3 ± 3503.9*	4323.7 ± 685	14580.2 ± 2802.1**
Beta	4023.3 ± 1266.1	7459.3 ± 1611.2**	6910.1 ± 1481.1	12120.3 ± 3167.5*	3233.7 ± 536.3	11932.6 ± 2267.9**
Gamma	2717.3 ± 800.6	5646.2 ± 1135.4**	4761.9 ± 1035.3	9075.9 ± 2473.2*	2535.6 ± 454	9598 ± 1794.9**

The average spectral amplitude ± SEM (pA/Hz) is shown before (control) and during orexin action (Orexin-A) for key frequency bands. Groups were compared with paired *t*-tests.

*p < 0.05.

**p < 0.01.

### Does orexin-mediated high frequency noise enhance calcium-dependent resonances in LDT and PPT cholinergic neurons?

**Figure 8:**
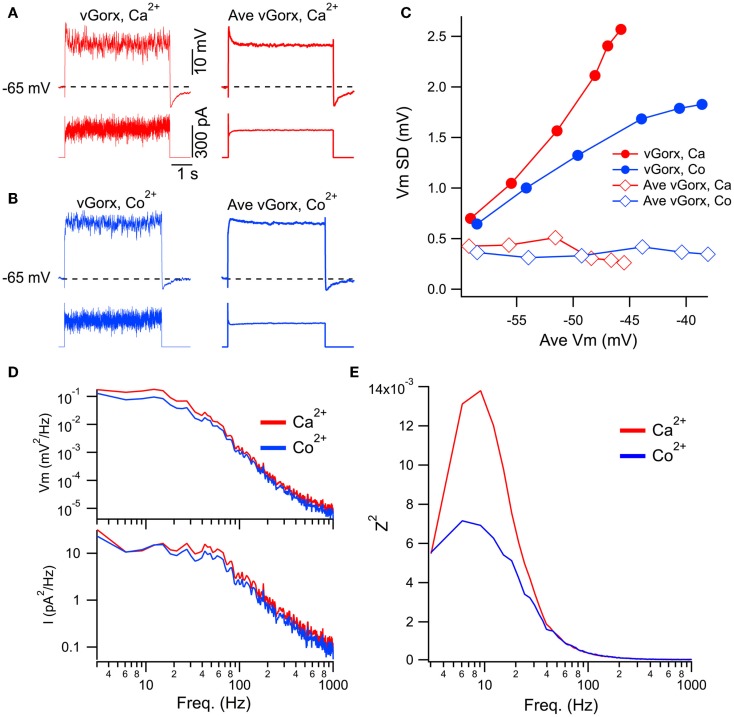
**Dynamic clamp input of a noisy virtual orexin conductance (vGorx) reveals an intrinsic Ca^2+^ dependent resonance**. **(A)** Sample recording from a cholinergic LDT neuron in normal Ca^2+^ ACSF (Ca^2+^) of membrane potential (top row) in response to injected current (bottom row) produced by an 8 nS vGorx with noise (vGorx; left traces) and without noise (Ave vGorx; right traces). Comparable average depolarizations were produced by the conductance with and without noise. **(B)** Identical recordings from the same neuron after blocking Ca^2+^ channels with Co^2+^-ACSF (Co^2+^). **(C)** Fluctuations in Vm (Vm SD) as a function of the average depolarization achieved by different magnitudes of vGorx. Filled symbols indicate fluctuations produced by the noisy conductance (vGorx) and open symbols indicate fluctuations produced by Gorx without noise (Ave vGorx). **(D)** Power spectral density of Vm (top) and input current (bottom) in normal Ca^2+^ ACSF (Ca^2+^, red) and Co^2+^ ACSF (Co^2+^, blue) resulting from 8 nS vGorx. Note the double log scales. **(E)** The impedance squared (Z^2^) reveals a broad resonance peaking between 6 and 12 Hz in response to 8 nS vGorx in Ca^2+^ ACSF (red). Blocking Ca^2+^ channels with Co^2+^ ACSF strongly attenuated this resonance (blue).

The data above indicate that in addition to a slow depolarization, orexin receptor activation provides substantial high frequency input to mesopontine cholinergic and serotonergic neurons. Such a broad-band input might be expected to drive any intrinsic resonances that might exist within these neurons. As described in the Introduction, an intrinsic Ca^2+^-dependent gamma band oscillation has been described in PPT neurons. In the final series of experiments, we utilized a dynamic clamp to test the ability of a noisy virtual orexin conductance (vGorx) to drive Ca^2+^ dependent resonances in these neurons. To do so, we applied different magnitude vGorx in normal Ca^2+^ ACSF and then again in ACSF with Co^2+^ substituted for Ca^2+^ to block Ca^2+^ channels. Since such resonances may be remote from the cell body and might be activated well above spike threshold, TTX was added to the ACSF to block voltage-gated Na^+^ currents in order to focus on Ca^2+^-dependent responses. An example of this approach for a cholinergic LDT neuron is illustrated in Figure 8. The neuron was driven with a vGorx (Figure 8A, left) and then again by an equivalent vGorx without the noise (Figure 8A, right, Ave vGorx) in ACSF with normal Ca^2+^ (red traces) and in ACSF with Co^2+^ substituted for Ca^2+^ (Figure 8B, blue traces). We next compared the fluctuations in the voltage response as a function of the average membrane potential achieved by the input of different magnitude vGorxs (Figure 8C). This analysis revealed that voltage fluctuations, measured as the standard deviation of Vm (Vm SD), grew steeply with the achieved depolarization (and increased vGorx) when noise was present (filled symbols) but not when the cell was driven by just the average vGorx (i.e., without the conductance fluctuations; open symbols). This indicates that the depolarization alone did not drive Ca^2+^ spiking or the large increase in voltage fluctuations, and that the increase in voltage fluctuations requires the noisy input. To assess the contributions of Ca^2+^ dependent intrinsic processes, we then examined the voltage responses to the same conductance inputs with Co^2+^ ACSF (Figure 8C, blue symbols). This revealed that the voltage fluctuations produced by the noisy vGorx grew with the achieved depolarization (and increased vGorx) but did so more slowly than in Ca^2+^ ACSF. This suggests that voltage-dependent Ca^2+^ channels and/or other Ca^2+^-dependent processes boost the voltage responses to vGorx.

To examine the frequency-dependence of these processes, we computed the ratio (impedance squared, Z^2^; Figure 8E) of power spectra from the voltage response (Figure 8D, upper; Vm^2^) and the input current (Figure 8D, lower; I^2^). This revealed a broad resonance peaking between 6 and 12 Hz in response to vGorx (8 nS) in normal Ca^2+^ ACSF (Figure 8E, red trace). This resonance was strongly attenuated in Co^2+^ ACSF, indicating an intrinsic Ca^2+^-dependent process, presumably voltage-gated Ca^2+^ channel activity, drove it.

**Figure 9:**
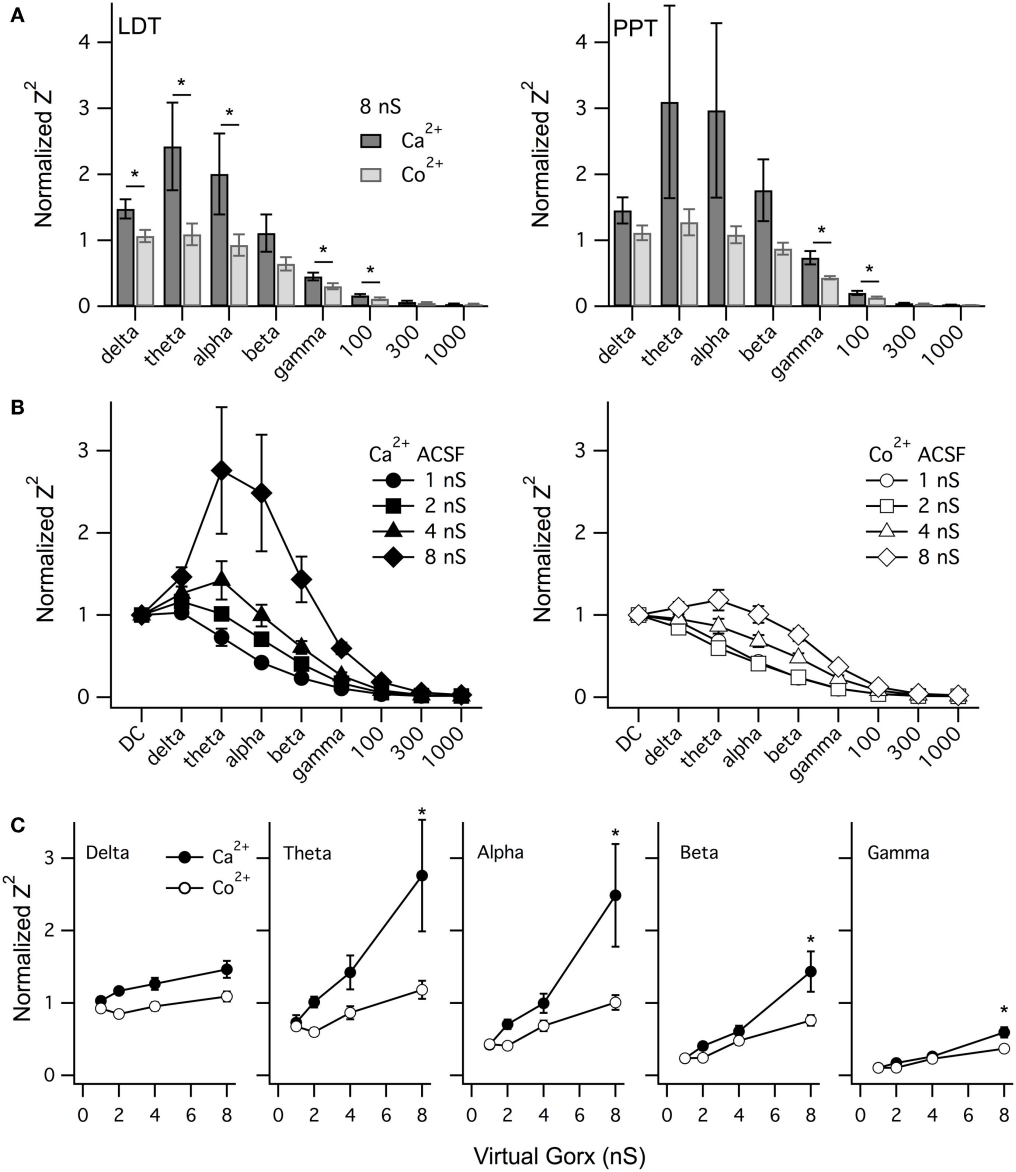
**vGorx evoked a broad Ca^2+^-dependent resonance in LDT and PPT cholinergic neurons**. **(A)** The average (±SEM) of the normalized impedance squared (Normalized Z^2^) was compared across neurons for key frequencies measured from LDT (left) and PPT (right) cholinergic neurons during an 8 nS vGorx input. In LDT neurons, the average normalized Z^2^ (black) was significantly reduced by Co^2+^ (gray) in the delta, theta, alpha, gamma, and 100 Hz frequency bands (*p* < 0.05). Variability was greater for PPT neurons but the same pattern was manifest, although statistical significance was only reached for the gamma and 100 Hz bands (*p* < 0.05). **(B)** The resonance measured in Ca^2+^ ACSF (Ca^2+^, left panel) grew with vGorx and was greatly attenuated in Co^2+^ ACSF (Co^2+^, right panel). **(C)** The effect of vGorx on normalized Z^2^ at delta, theta, alpha, and gamma frequency bands in both Ca^2+^ and Co^2+^ ACSF. (see Results for details). Frequency bands were defined as delta: 1–4 Hz; theta: 4–8 Hz; alpha: 8–14 Hz; beta: 14–30 Hz; and gamma: 30–60 Hz.

To compare the effect of vGorx across neurons, we measured Z^2^ at key frequencies for LDT (*n* = 6) and PPT (*n* = 6) neurons and normalized it to the corresponding DC value of Z^2^. Figure 9A shows the average normalized Z^2^ (±SEM) at each band produced by vGorx (8 nS) in both control conditions (Ca^2+^ACSF) and after superfusing with Co^2+^ (Co^2+^ ACSF). A similar pattern of Ca^2+^ dependent resonance was seen in LDT and PPT cholinergic neurons. In the LDT, Co^2+^ reduced the average normalized Z^2^ in the delta, theta, alpha, gamma, and 100 Hz frequency bands (*p* < 0.05). The variability was greater among PPT neurons, largely due to one neuron having an unusually large Ca^2+^ dependent resonance, and the reduction produced by Co^2+^ achieved significance only at the gamma and 100 Hz frequency bands (*p* < 0.05).

We next examined how this resonance varied with the strength of vGorx. Since the resonance was similar in both LDT and PPT cholinergic neurons, we pooled data from both nuclei. Figure 9B illustrates the dependence of normalized Z^2^ on vGorx in Ca^2+^ ACSF (left panel) and in Co^2+^ ACSF (right panel). It was clear that both the magnitude and the width of the resonance increased with increasing vGorx. The resonance peaked in the theta range for inputs of 4 and 8 nS (*n* = 12). A 12 nS vGorx was delivered to four neurons (three PPT and one LDT), and the resonance was larger and broader and peaked in the alpha frequency range. Two neurons (one PPT and one LDT) were also tested with a 16 nS vGorx and the resonance was yet larger and wider and peaked in the alpha band of frequencies. All of these resonances were powerfully attenuated by switching to Co^2+^ ACSF (right panel).

To compare how this Ca^2+^-dependence varied with the strength of vGorx, we plotted the normalized Z^2^ vs. vGorx at delta, theta, alpha, beta, and gamma bands in both Ca^2+^ and Co^2+^ ACSF (Figure 9C). For each frequency band (including 100 Hz), there was a significant effect of vGorx on Z^2^ (repeated measures MANOVA, *p* < 0.0001). Similarly, for each frequency band, Z^2^ was significantly lower in Co^2+^ ACSF (repeated measures MANOVA, *p* < 0.01). In addition, for the theta, alpha, beta, gamma, and 100 Hz frequencies, there was a significant interaction between vGorx and the treatment with Co^2+^ (repeated measures MANOVA, *p* < 0.05). *Post hoc* testing revealed that for each frequency band, the normalized Z^2^ produced by 8 nS vGorx was significantly reduced in Co^2+^ ACSF (*p* < 0.001, least squared differences).

Collectively, our dynamic clamp findings demonstrate that broad-band noise from a virtual orexin conductance activates a Ca^2+^ dependent resonance in cholinergic LDT and PPT neurons that peaks at frequencies much lower than gamma, in the theta and alpha frequency range (4–14 Hz). The resonance was, nonetheless, rather broad with the impedance up to 100 Hz reduced in Co^2+^ ACSF.

## Discussion

The main findings of this study are: (1) ChAT-Cre mice are highly suitable for identifying and manipulating LDT and PPT cholinergic neurons; (2) FEV-Cre driven expression of dTom can serve as a good marker for most medium to large TPH + DR neurons; (3) independently of any presynaptic actions of orexin, noise from the post-synaptic orexin current provides substantial high-frequency input to cholinergic neurons of the LDT and PPT, and serotonergic neurons of the DR; (4) Cholinergic LDT and PPT neurons have a broad Ca^2+^ dependent intrinsic resonance that peaks in the theta and alpha range of frequencies, and is effectively engaged by the high-frequency components of the noisy orexin current.

### Reporter accuracy in ChAT-Cre and FEV-Cre mice

The expression pattern of reporter fluorescence in the offspring of ChAT-IRES-Cre mice followed the pattern of ChAT immunofluorescence in the LDT and PPT with high fidelity, showing both excellent efficacy and specificity. In the first cohort analyzed, we counted a lower fraction of ChAT+ neurons expressing EYFP than in the second cohort (Figure 1). While this difference might reflect reporter efficiency, it is more likely to reflect differences in our analysis. In the first cohort, image exposure was chosen to avoid saturating the brightest ChAT+ and EYFP+ neurons. This risks underexposing neurons with the weakest fluorescence, and it was the most weakly labeled ChAT+ neurons that appeared negative for EYFP. In the second cohort, reporter fluorescence (ChR2-EYFP) was closely associated with the membrane and we examined multiple exposures of each field to ensure we could detect this label. In these images, we were able to see membrane associated EYFP fluorescence, even in faintly labeled ChAT+ neurons. Thus, false negative EYFP label was more likely in the first cohort, suggesting we underestimated the number of EYFP positive neurons.

The overall high fidelity and specificity of reporter fluorescence in ChAT+ neurons was consistent with previous observations in preganglionic parasympathetic and sympathetic neurons (28). Moreover, it was consistent with our expectations based on the approach used to engineer these mice. Cre expression is driven by the endogenous ChAT gene since the IRES-Cre sequence was inserted following the endogenous ChAT stop codon (28). This avoids positional effects that can account for variable reporter expression following random transgene insertion. For example, we analyzed a transgenic ChAT-Cre mouse strain (MMRRC 030869-UCD) that was crossed with the same EYFP reporter used here and we found much lower fractions of ChAT+ LDT/PPT neurons expressing Cre-induced fluorescence (<20% in LDT and PPT, *n* = 3), even though nearly 100% of oculomotor and trochlear motorneurons expressed fluorescence (Kang and Leonard, unpublished observations). Use of the ChAT-IRES-Cre mice also avoids a problem discovered with recent BAC transgenics developed for visualizing (ChAT-GFP) and manipulating (ChAT-ChR2-EYFP) cholinergic neurons, where additional copies of the vesicular ACh transporter (VAChT) were introduced in the BAC construct. This results in over-expression of VAChT in these mice and a hypercholinergic phenotype (43–45). Thus, ChAT-IRES-Cre mice are highly suitable for studies in which LDT/PPT cholinergic neurons need to be visualized or manipulated with Cre/Lox approaches.

In the DR, previous studies have illustrated the utility of FEV-Cre mice for marking and manipulating serotonergic neurons. In these mice, an upstream enhancer region (ePet) for the Pet-1 gene, which is transcribed selectively in serotonergic neurons, is exploited to control Cre expression (29). Although offspring of these FEV-Cre and dTom reporters has previously been used to label and manipulate serotonergic neurons (46, 47), those studies did not quantify expression accuracy. Here, we found (Figure 2) that medium to large TPH+ neurons were accurately labeled but that only 60–70% of these TPH+ neurons were labeled. Moreover, a variable population of small neurons with undetectable TPH immunofluorescence was observed in the DR and appeared most prevalent in the dorsal sector of the dorsal DR in these mice. Future studies will be necessary to determine the nature of this population and if it is also labeled with other reporters.

### High-frequency input from orexin receptor activation

Orexin peptides produce a slow depolarization that is mainly mediated by a noisy cation current in LDT, PPT, and DR neurons (22–27). Here, we analyzed the spectral composition of this noisy current in identified cholinergic LDT and PPT neurons and serotonergic DR neurons. We found that the orexin current noise added significant power at high frequencies including gamma frequencies, but that the noise was broad-band rather than being concentrated into one or more narrow bands. This is consistent with the noise being generated by the random opening and closing of the ion channels gated by orexin receptor activation. Currently, the molecular identity of these channels has not been elucidated. A direct comparison of LDT and DR neurons (25) indicated that while a near-maximal concentration of orexin induced a current that was ~2–3 times larger and noisier in DR neurons, the currents appeared similar, in that they were both Cs-permeable, blocked by lowering extracellular Na^+^, relatively impermeable to Ca^2+^ ions, and had similar I–V relations. Nevertheless, some differences were noted. For example, in low-Ca^2+^ ACSF, the DR current was significantly augmented while the LDT current was not. Moreover, the noisy current was activated by both OX_1_ and OX_2_ receptors in DR neurons but was only activated by OX_1_ receptors in LDT neurons, even though functional OX_2_ receptors appeared to be present in these neurons (27). Thus, in spite of similarities, it is possible that different channels mediate the orexin-activated noisy cation current in these neurons. Presumably, these channels are of the TRP superfamily, which make many types of cation currents [for review, see Ref. (48)], and TRPC subfamily subunits are expressed in serotonergic DR neurons (49). However, the pharmacological tools to dissect TRPC channels containing particular subunits are poor. Additional insight into the underlying channel properties, such as single channel conductance, number of channels, and probability of opening can be gleaned using fluctuation analysis [for review, see Ref. (50)]. While this approach has been applied to some neurons in slices (51), we have deferred this analysis until we can clarify channel location, since a dendritic localization would introduce large errors into the analysis.

Neural systems are intrinsically noisy with major noise sources arising from random ion channel gating and synaptic input [for review, see Ref. (50, 52)]. While noise is typically thought about in terms of degrading system performance and reliability, it is clear that under some circumstances it can be helpful. A well-known example of this, which occurs in non-linear systems with a threshold, is the process termed stochastic resonance (SR) (50, 53). In SR, addition of an optimal amount of noise greatly improves the signal-to-noise ratio for weak signals and enables subthreshold input, which alone would have no impact, to become effective in driving output. We therefore propose that one function of orexin current noise is to enhance the effectiveness of synaptic inputs to these neurons by SR. A related and potentially beneficial function of this noisy input can be anticipated based on the study of balanced inhibitory and excitatory synaptic inputs, which modulate the slope of the input–output relation (54, 55). The noisy orexin current could work in a similar manner by providing both the necessary input fluctuations and conductance change. Future investigations will be necessary to examine this possibility.

Under physiological conditions, orexin neurons initiate firing before, and have firing rates that are highest during, periods of active waking. Moreover, firing rate is positively correlated with gamma components and negatively correlated to lower frequencies of the EEG (56, 57). This suggests that orexin release would be likely to occur before and during active waking states rich in gamma. Slice studies of orexin input to tuberomammillary histamine neurons indicate glutamate and orexin are co-released, but that during persistent firing, glutamate synaptic activity depresses while orexin excitation grows with stimulus duration (58). These findings suggest that the synaptic release of orexin is well mimicked by bath application. If orexin synaptic inputs to LDT, PPT and DR neurons behave similarly, the magnitude of the post-synaptic depolarization would reflect the integrated activity of the orexin inputs, while the noisy effectors in these neurons would recapitulate the high frequency components of orexin input firing. Such a combined signal would be well-suited to enhance excitability of cholinergic and serotonergic neurons during active waking.

### Cholinergic LDT and PPT neurons have a broad Ca^2+^-dependent resonance that peaks at theta and alpha band frequencies

Many neurons have intrinsic resonances, and an effective way to elicit and study these resonances is with an input containing a broad range of frequencies, like that of the orexin current noise [for review, see Ref. (59)]. We found that in cholinergic LDT and PPT neurons, membrane impedance developed a clear peak that increased with the magnitude of vGorx. This peak was eliminated in Co^2+^ ACSF (Figures 8 and 9), which also suppressed the impedance up to 100 Hz (Figure 9). Resonances result from the interplay between the active and passive membrane properties and these data strongly indicate that high-threshold, voltage-gated Ca^2+^ channels play an active role in generating the resonances in cholinergic LDT and PPT neurons. Future experiments will be necessary to identify the Ca^2+^ channels involved. Previous studies of PPT neurons indicate that P/Q-type channels are necessary for the ramp-evoked gamma oscillations (60). In our prior study of cholinergic LDT neurons, we found that L-, N-, and R-type voltage-gated Ca^2+^-channels all contribute to spike-evoked Ca^2+^ influx, but we found no evidence for P/Q-type channels in the soma or proximal dendrites of these neurons (61). This would suggest that P/Q channels are not necessary for the resonances observed here. However, our study focused on channels contributing to the somatic and proximal dendritic Ca^2+^ influx; so, it remains possible that P/Q channels are differentially distributed to the distal dendrites, and that perhaps these or other distally localized channels are necessary for the resonance. Indeed, electrotonically remote P/Q channels have been suggested to explain the strong somatic depolarization required to elicit gamma frequency oscillations in PPT neurons (60). However, high speed imaging results now indicate that corresponding Ca^2+^ oscillations are present at both the soma and proximal dendrites, consistent with a more uniform distribution of these channels (62). This is also consistent with our prior findings in LDT cholinergic neurons, where we found N- and R- channels in both the soma and proximal dendrites, although L-type channels appeared localized to the soma (61).

It is noteworthy that over the conductance range we tested, vGorx did not induce large-amplitude gamma frequency oscillations like those produced by strong ramp depolarizations. Those oscillations emerge at more depolarized potentials (60), and while stronger depolarizations might have produced such oscillations, the estimated reversal potential of the orexin current and vGorx (−15 mV) is a barrier to substantially stronger depolarizations derived from this conductance mechanism. Another consideration is that our dynamic clamp recordings were conducted at 32°C. While high-threshold gamma band oscillations occur even at 30°C (63), future experiments will be needed to evaluate both the post-synaptic resonance identified here and the orexin noise at physiological temperature.

### Functional considerations for the Ca^2+^-dependent resonance in cholinergic LDT and PPT neurons

Intrinsic resonances increase response selectivity of neurons by amplifying inputs within the frequency band of the resonance. Our dynamic clamp experiments show that cholinergic LDT and PPT neurons have a broad resonance that can be effectively driven by the spectral properties of orexin current noise. We expect that other broad-band inputs, like fast synaptic noise from random EPSPs and IPSCs, will also activate this resonance and that inputs driven at discreet frequencies within the resonance band will also be amplified. The Ca^2+^ dependence to the impedance extended up to 100 Hz, indicating the resonance affects a wide range of frequencies, but the resonance peaked in the theta (4–8 Hz) frequency range for 4–8 nS inputs and in the alpha range (8–14 Hz) for stronger inputs (12 and 16 nS inputs). Thus, while we expect this resonance to boost gamma frequency synaptic inputs, we expect a much greater boost to synaptic inputs occurring at theta and alpha frequencies.

A critical consideration in assessing the functional importance of resonances is how they interact with the spike generation mechanisms. Subthreshold resonances, which can result in oscillations, facilitate the encoding of resonance-amplified inputs into action potentials (59). Such resonances have been illustrated for many types of neurons with peaks occurring over frequencies ranging from below 1 Hz to greater than 40 Hz (10, 64–70).

It remains to be determined how the Ca^2+^-dependent resonance, described here, interacts with the spiking mechanism, since spiking was blocked with TTX for these experiments. Our data suggest that the resonance would be active at membrane potentials below spike threshold (Figure 8C, see difference between filled symbols), which we expect to be between ~−45 and −53 mV (30, 42, 71), and could therefore influence subthreshold membrane potential behavior. These neurons also have TTX-sensitive subthreshold oscillations that can influence spiking, especially around theta frequencies (30, 42). Thus, it is possible that subthreshold activation of sodium channels works with the Ca^2+^ dependent resonance to help encode suitable synaptic frequencies into action potentials.

Such an encoding role is also consistent with the firing rates of cholinergic LDT and PPT recorded *in vivo*. Studies in unanesthetized rodents indicate that these neurons fire at their highest rates during active waking and REM sleep where they reach average firing frequencies of ~2–4 Hz (72, 73). These behavioral states are characterized by an EEG rich in theta and higher frequencies including gamma. The firing of these neurons was positively correlated with these EEG epochs, although firing was not rhythmic and was not phase-locked to these rhythms, even though these cells could fire at high enough rates, in bursts and spike clusters. This has also been reported in the anesthetized rat, where cholinergic PPT neurons fire during periods of nested cortical gamma activity but don’t fire at gamma rates and are not phase locked to gamma activity (74). Thus, if intrinsic high-threshold gamma oscillations are naturally occurring in these neurons, they are not being encoded into gamma frequency firing. Rather, we propose that the noisy orexin input and the post-synaptic Ca^2+^ dependent resonance, described here, work synergistically to boost responsiveness to synaptic inputs occurring mainly in theta and alpha bands but extending to higher frequencies. This will enhance integration of synaptic inputs at these frequencies and promote their encoding into action potentials. However, once an action potential occurs, the ensuing currents, including the AHP-prolonging A-type, and Ca^2+^ activated K^+^ currents, will limit firing rate and disrupt the propensity to phase-lock at these frequencies. Nevertheless, the resonance will still help ensure that firing of mesopontine cholinergic neurons increases during periods of cortical activation so that their output can reinforce thalamocortical states supporting cortical gamma and its associated functions.

## Conflict of Interest Statement

The authors declare that the research was conducted in the absence of any commercial or financial relationships that could be construed as a potential conflict of interest.

## Footnotes

^1^imagej.nih.gov/ij/

^2^kurt.devos@iop.kcl.ac.uk
